# Predictors of Pharmacy Students' Attitudes About the Therapeutic Use of Psilocybin

**DOI:** 10.7759/cureus.45169

**Published:** 2023-09-13

**Authors:** NM Mahmudul Alam Bhuiya, Robin J Jacobs, Karina Wang, Yiqun Sun, Brenda Nava, Luke Sampiere, Akhila Yerubandi, Joshua Caballero

**Affiliations:** 1 Pharmacy, University of Georgia, Athens, USA; 2 Dr. Kiran C. Patel College of Osteopathic Medicine, Nova Southeastern University, Fort Lauderdale, USA

**Keywords:** ocd/anxiety disorders, depression, magic mushroom, mental health, psychiatric, alternative medicine, palliative care, pharmacy education, legalization, schedule 1 drug

## Abstract

Background

Psilocybin has been studied for its potential therapeutic benefits, particularly for the treatment of psychiatric disorders such as anxiety, depression, and obsessive-compulsive disorder. While more research is needed as psilocybin-assisted therapy becomes more prevalent, future pharmacists will probably be involved at some level. At present, pharmacists receive minimal training on psilocybin, and little is known about their attitudes toward its use for medical purposes. Findings from recent clinical studies have attempted to establish the safety and medical efficacy of psilocybin, leading to an increased interest in therapeutic psilocybin use in the United States. This study aimed to assess if self-assessed knowledge of psilocybin, concerns about adverse effects, and opinions about legalization will make statistically significant contributions to pharmacy students' attitudes about psilocybin use in practice.

Methods

Pharmacy students’ self-assessed knowledge, concern for potential adverse effects, and perceptions of psilocybin were investigated using a cross-sectional survey study design. Data were collected from March 13 to April 7, 2023, from a convenience sample of 161 pharmacy students enrolled in an accredited pharmacy school in the southern region of the United States using a 41-item anonymous quantitative survey developed by the researchers that contained validated scales. The survey was delivered electronically. Multiple regression modeling was conducted to determine if self-assessed knowledge, concerns for adverse effects, and opinions about legalization would predict pharmacy students’ attitudes about therapy-assisted psilocybin use. This study was approved by the Institutional Review Board of the authors' university.

Results

The mean age of the 161 participants was 24 years (SD = 2.981; range 20-40 years). Twenty (12.4%) participants reported previous use of psilocybin for recreational purposes and two (1.2%) reported having used it therapeutically. Many (n =121; 75.2%) of the participants believed that psilocybin should be decriminalized for therapeutic use, but only 54 (33.5%) thought it should be decriminalized for recreational use. A multiple linear regression model predicting "attitudes about psilocybin" (dependent variable) produced significant results: (F(4, 122) = 40.575, p < 0.001), with an R2 = 0.571 (adjusted R2 = 0.557). Greater "self-assessed knowledge about psilocybin," less "concern about possible negative effects," greater "belief in the decriminalization of psilocybin for recreational use," and greater "belief in the decriminalization of psilocybin for therapeutic use" (all independent variables) were associated with more positive perceptions about medical psilocybin. The percentage of variance in the scores accounted for by the model was 57%.

Conclusions

Pharmacy students may lack information and training regarding psilocybin and report a desire to learn more about it. Their attitudes about medical psilocybin may be driven by this desire to learn in addition to concerns about adverse effects and legalization issues. Due to the dearth of published information regarding the knowledge and acceptance of psilocybin as a viable treatment option for patients, further research in psychedelic-assisted treatments may be warranted.

## Introduction

Psilocybin, the active ingredient in certain varieties of hallucinogenic mushrooms, which are commonly known as "magic mushrooms," has long been utilized as a psychedelic substance in religious and spiritual rituals and as a treatment for neuropsychiatric disorders in recent times [1-3]. The term "psychedelics" (serotonergic hallucinogens) refers to a class of "powerful psychoactive substances that alter perception and mood and affect numerous cognitive processes" [4,5]. Psilocybin's potential therapeutic (i.e., medical) benefits, particularly for the treatment of mental health disorders (e.g., depression and anxiety) have drawn increasing attention in recent years [6-13]. In human laboratory and clinical trial studies, psilocybin has been shown to have a physiological and psychological safety profile and to be non-addictive in addition to possessing anti-addictive characteristics [14,15].

Legalization issues

Psilocybin is categorized as a Schedule I controlled drug under the Controlled Substances Act [16]. Schedule I drugs are substances with a high potential for abuse and no currently recognized medicinal value. However, findings from recent studies in humans and animals, including a large multi-site clinical trial with psilocybin for treatment-resistant depression, have attempted to establish the safety and medical efficacy of psychedelics, leading to an increased interest in therapeutic psilocybin in the United States [17,18]. In 2019, psilocybin use and possession were legalized in parts of Colorado, California, and Michigan [19]. Additionally, the United States Food and Drug Administration has designated psychedelics (e.g., psilocybin) as "potential treatments," even offering draft guidance for clinical trials evaluating the future safety and effectiveness of these agents [20].

Safety and adverse effects

Of all the psychedelic substances, psilocybin is considered the safest [21], and some healthcare professionals have reported confidence in psychedelic-assisted therapy within properly controlled settings [22]. However, although psilocybin has been reported to be relatively safe and can result in positive experiences under a controlled environment, there can be some negative consequences such as transient paranoia, nausea, and headaches lasting several days [23]. However, harm-reduction approaches need to be considered when administering psilocybin-assisted therapy to patients [24].

Knowledge and attitudes about psilocybin among pharmacists

To our knowledge, there are few published reports of the perceptions, knowledge, and attitudes of healthcare professionals in general and pharmacists and/or trainees regarding psilocybin-assisted therapy [25-27]. Mental health professionals tend to have unfavorable attitudes and minimal self-rated expertise in psilocybin [28]. Findings from a study assessing medical students' perceptions of psilocybin-assisted therapy indicated that medical trainees who hold more favorable attitudes toward psilocybin-assisted therapy tend to have less concern for adverse events, greater knowledge about psilocybin, and greater belief in psilocybin legalization for recreational (not therapeutic) use [25].

Pharmacists' knowledge of the therapeutic use of psilocybin remains limited. A survey study conducted with pharmacists reported that 73% of respondents lacked formal education in psilocybin-assisted therapy and 45% reported they were uncomfortable with laws pertaining to psilocybin [29]. Moreover, the participants were not comfortable with monitoring or dosing, and only 37% thought psilocybin was a useful alternative to conventional medicine [29].

A study investigating the attitudes, knowledge, and awareness of medical cannabis use among pharmacy students explored the possible utility of integrating medical cannabis education into pharmacy curricula [30]. Similar to marijuana, the legalization of psilocybin may become a legally prescribed medication; future pharmacists may need to have the requisite practical expertise and knowledge in this area. However, due to the varying legal status of psilocybin from state to state (including issues regarding liability and compliance with professional standards), pharmacists may need to be kept informed about legal and regulatory approval.

Pharmacists advise patients on how to take medications safely and effectively [31]. As one of the most accessible healthcare providers, community pharmacists, in particular, are well positioned to provide education and counseling to patients on the safety and efficacy of psilocybin, yet few studies exist exploring pharmacists' knowledge, experiences, and attitudes toward it. Future pharmacists’ perspectives on the issue could shape how the profession embraces or rejects the idea and may drive curricular efforts.

Research questions

The researchers of the current study have published reports about the perceptions of both medical cannabis and psilocybin-assisted therapy among medical students [25,32-34]. However, little is known about pharmacy students’ attitudes toward psilocybin, including their personal experiences with it. This study thus asked the following questions: (1) What are pharmacy students’ self-assessed level of knowledge, beliefs, concerns about negative effects, and overall attitudes toward psilocybin’s use as a treatment? (2) Will self-assessed knowledge, beliefs, and concerns influence their overall attitudes about its role in the treatment of various disorders? It was hypothesized that pharmacy students’ self-assessed knowledge, concern about negative effects, and beliefs about the legalization of psilocybin will make statistically significant contributions to their perceptions toward psilocybin use in pharmaceutical practice.

## Materials and methods

Study design and sample

A survey study with a cross-sectional design was carried out from March 13 to April 7, 2023, to assess pharmacy students' knowledge and attitudes toward psilocybin use for therapeutic purposes, herein referred to as the Pharmacy Students Perceptions of Psilocybin (PSPP). Using a convenience sample of pharmacy students enrolled in an accredited pharmacy program, study data were collected and managed using REDCap electronic data capture tools (Vanderbilt University, Nashville, TN) [35]. Pharmacy students were contacted via student email listservs and invited to participate. They were informed of the voluntary nature of the study and that participation would not impact their grades or academic standing within their pharmacy program. The University of Georgia Institutional Review Board approved this study (protocol number: PROJECT00006889).

Instrument

The PSPP was developed by the researchers and contained 41 items that investigated medical students’ attitudes toward psilocybin. The items for this instrument were adapted from the authors’ previous study about medical students' attitudes toward cannabis [32-34] and a follow-up study on their attitudes toward psilocybin [25]. The researchers developed the instruments after reviewing recent and relevant studies published in peer-reviewed journals as well as anecdotal reports from health professions educators. The aim of the development of the original 32-item questionnaire (created by the researchers) was to evaluate medical students’ attitudes, concerns about its possible adverse effects, and knowledge about medical cannabis. All items were checked for face validity through the agreement of three health professions educators (from medicine, pharmacy, and social work) and three medical students in various years of study. The items assessed for the medical cannabis study also investigated medical students’ opinions about legalization and prescribing.

Using principal component analysis with varimax rotation as an exploratory method, the factor structure of the instrument to determine medical students’ perceptions of medical cannabis was investigated. Three factors were identified: (1) attitudes toward medical cannabis, (2) concern for possible adverse effects of medical cannabis, and (3) perceived knowledge of medical cannabis. Factor 1 accounted for approximately 41.5% of the total variance; the reliability estimate (Cronbach’s α) for the first factor was calculated at 0.91. The second factor accounted for 9.5% of the variance and a reliability estimate of 0.89. Factor 3 accounted for 8.7% of the variance; the reliability estimate was calculated at 0.89 [34].

The researchers then adapted the medical cannabis instrument to assess the attitudes about psilocybin among medical students [25]. The results of this study showed that medical students with a greater self-assessment of their knowledge about medical psilocybin, less concern for its potential adverse effects, and the opinion that psilocybin should be federally legalized for recreational purposes have more positive attitudes about its medical use.

The PSPP, a 41-item survey that assessed perceived knowledge, concerns about possible negative effects, and attitudes about psilocybin in pharmacy students, was adapted from the original medical cannabis survey [32]. The PSPP used Likert-type items with a six-point response scale (1 = strongly agree to 6 = strongly disagree) and for consistency, reverse scored for the concern scale (1 = strongly disagree to 6 = strongly agree). Also included were categorical items and yes/no questions. Many of the items were adapted from various published studies, mostly regarding the use of medical cannabis, as no measures specific to medical psilocybin could be found. The content areas assessed and their scoring are delineated below.

Self-Assessed Knowledge of Psilocybin

To assess self-assessed knowledge of psilocybin, four Likert-type items collected data on participants’ self-assessed knowledge regarding the medicinal uses of psilocybin. Each item was rated using a six-point response set (1 = strongly agree to 6 = strongly disagree), with higher scores indicating higher levels of self-assessed knowledge about psilocybin [25,32,34]. The items in the scale are (1) "I have good knowledge of the side effects of psilocybin, (2) “I am familiar with the possible therapeutic effects of psilocybin,” (3) “I am extremely confident regarding my current knowledge of psilocybin," and (4) "I have substantial knowledge about psilocybin" [25,32,34].

Concern About Negative Effects

Four items were included to ascertain participants’ concern for possible negative effects of psilocybin use: (1) "I am concerned about the potential side effects of psilocybin use," (2) “Psilocybin use can be addictive, (3) “I am concerned with psilocybin’s potential for addiction or its psychoactive properties," and (4) "I am concerned with psilocybin’s potential for abuse or misuse." Each item was rated using a six-point response set (1 = strongly agree to 6 = strongly disagree), with higher scores indicating greater concern for possible negative effects of psilocybin use [25,32,34]. In addition, a single item was included that asked how concerned the participant was regarding the limited evidence of the therapeutic benefits from psilocybin use, scored on a numerical scale of 1 (least concern) to 7 (most concern) [25,32,34].

Attitudes About Psilocybin

Fourteen items assessed overall attitudes toward medicinal psilocybin. Sample items are: (1) "I believe that psilocybin has medical benefits/efficacy," (2) "Psilocybin has a role in palliative care,” and (3) "Psilocybin helps patients who suffer from chronic, debilitating medical conditions” [25,32,34]. Each item was rated using a six-point response set (1 = strongly agree to 6 = strongly disagree), with higher scores indicating more positive attitudes about psilocybin [25,32,34].

Utility of Psilocybin for Certain Medical Conditions

Participants were asked if they thought that psilocybin had therapeutic value for any or all of the 26 medical conditions or symptoms (e.g., anxiety, depression, insomnia, and alcohol use disorder).

Single Items

Demographic data were also collected (i.e., race, sex, Hispanic identity, and age in years), political views (i.e., liberal, independent, and conservative), current academic level in the pharmacy program, use of recreational and/or therapeutic psilocybin, if they knew any individuals who may have used psilocybin (recreationally and/or medicinally) in the past, and their thoughts about the legalization of psilocybin (for recreational and therapeutic use). Participants were also asked to identify medical conditions (from a list) they believed could be successfully treated with psilocybin, and if they were interested in learning more about psilocybin.

## Results

This study was conducted by inviting all enrolled University of Georgia pharmacy students in all four years of training (N = 557) to complete an electronic survey regarding their attitudes toward psilocybin. Of those, 225 students returned the survey (40% response rate). Of the 225 surveys, 64 surveys were eliminated due to having less than 66% of the items completed, resulting in 161 surveys included in the final analysis (71.6% completion rate).

IBM SPSS Statistics for Windows, version 28.0 (IBM Corp., Armonk, NY) [35] was used to conduct all analyses from data extracted from REDCap [36]. Reliability estimates (i.e., Cronbach’s alpha) for the three measures in the survey were calculated and all were within acceptable limits (α > 0.70) [37]: self-assessed knowledge (α = 0.92); concern for negative effects (α = 0.88); and attitudes toward psilocybin (α = 0.90). Multicollinearity testing was performed, and the independent variables (above) were within acceptable variance and inflation factor limits; all three measures were normally distributed.

Continuous variables such as age are reported as means and standard deviation (SD) and the discrete items such as the characteristics of the sample are displayed as frequencies and percentages. The point-biserial correlation coefficient was used due to the nature of the data, as it is a measure of the strength of association between the Likert scale scores (which in this case can be considered interval data) and binary variables (i.e., yes/no). Mathematically, the point-biserial correlation coefficient is calculated just as Pearson’s bivariate correlation coefficient would be calculated (wherein the dichotomous variable of the two variables is either 0 or 1). A correlation matrix was calculated to identify associations between only those independent variables that were hypothesized to be associated with the dependent variable (attitudes about psilocybin), after which multiple regression analysis was used for final hypothesis testing using those independent variables correlated with the dependent variable (which, in fact, turned out to be all of them). Multiple linear regression was performed using the "enter" approach whereby all the independent variables that were reported to be statistically significantly correlated with the dependent variable ("attitudes about psilocybin") were entered into one block.

Sample characteristics

The mean age of the participants in this sample (N = 161) was 24 years (SD = 2.98; range = 20-40 years). The majority (n = 146; 90.7%) were in the pre-clinical phase of training; the rest were in the clinical phase (n = 15; 9.3%). Characteristics of the sample are reported in Table 1.

**Table 1:** Characteristics of the sample

Characteristics	Frequency (n)	Percent (%)
Sex		
Woman	106	65.8
Man	54	33.5
Prefer not to answer	1	0.6
Academic status		
Pharm preclinical (years 1-3)	146	90.7
Pharm clinical (final year 4)	15	9.3
Race		
White	102	63.4
Black	21	13.0
Asian or Pacific Islander	28	17.4
Missing/declined	10	6.2
Cultural Identity		
Hispanic	8	5.0
Non-Hispanic	147	91.3
Prefer not to answer	6	3.7
Political view		
Conservative	42	26.1
Liberal	64	39.8
Independent	20	12.4
Other	12	7.5%
Prefer not to answer	23	14.3
Psilocybin use		
Previous use of psilocybin recreationally		
Yes	20	12.4
No	137	85.1
Prefer not to answer	4	2.5
Previous use of psilocybin therapeutically		
Yes	2	1.2
No	155	96.3
Prefer not to answer	4	2.5
Psilocybin should be decriminalized (made legal) in the United States for therapeutic use		
Yes	121	75.2
No	17	10.6
Prefer not to answer	23	14.3
Psilocybin should be decriminalized (made legal) in the United States for recreational use		
Yes	54	33.5
No	80	49.7
Prefer not to answer	27	16.8
Desire to learn		
Want to learn more about the therapeutic uses of psilocybin		
Yes	151	93.8
No	9	5.6
Prefer not to answer	1	6

Table 2 reports the findings from the item that asked participants to check any or all of the medical conditions they thought could be treated with psilocybin.

**Table 2:** Opinions about psilocybin’s therapeutic value for different health conditions and behaviors

Medical condition	Frequency (n)	Percent (%)
Alzheimer’s disease	50	31.1
Arthritis	26	16.1
Cancer	64	39.8
Chronic pain	80	49.7
COVID-19 (coronavirus)	2	1.2
Eating disorders	38	23.6
Fibromyalgia	32	19.9
Glaucoma	7	4.3
HIV/AIDS	10	6.2
Inflammatory bowel disease	9	5.6
Insomnia	46	28.6
Depression	96	59.6
Anxiety/post-traumatic stress disorder/obsessive-compulsive disorder	105	65.2
Other psychiatric illnesses (schizophrenia, bipolar disorder)	80	49.7
Migraines	47	29.2
Multiple sclerosis	17	10.6
Parkinson's disease	41	25.5
Persistent muscle spasm	18	11.2
Seizure/epilepsy	41	25.5
Palliative care	69	42.9
Alcoholism	32	19.9
Smoking	29	18.0
Phantom limb pain	37	23.0
Cluster headaches	30	18.6
Neurodevelopmental disabilities (autism, attention-deficit/hyperactivity disorder)	42	26.1
Other	11	6.8

Major study variables

Summary statistics (median and range) for the major study variables (scales) are reported in Table 3.

**Table 3:** Summary statistics of major study variables (scales) The self-assessed knowledge of the psilocybin scale and the attitudes about the psilocybin scale were structured using a six-point Likert-type response set, whereby 1 = strongly agree and 6 = strongly disagree. For consistency, the concern for negative effects of the psilocybin scale was reverse scored, whereby 1 = strongly disagree and 6 = strongly agree. For the self-assessed knowledge scale, psilocybin attitudes scale, and concern for negative effects of psilocybin scale, higher scores indicate greater self-assessed knowledge, more positive attitudes about psilocybin, and greater concern for negative effects, respectively.

Scale	n	Median
Self-assessed knowledge of psilocybin	161	4.25
Concern for possible negative effects	161	4.25
Attitudes about psilocybin	161	2.79

Table 4 reports the results of the correlation analysis for preliminary hypothesis testing. Only the variables that were statistically significantly correlated with attitudes toward psilocybin are reported.

**Table 4:** Correlations between attitudes about psilocybin (dependent variable) and independent variables ** Correlation is significant at the 0.01 level (two-tailed). * Correlation is significant at the 0.05 level (two-tailed).

	Attitudes about psilocybin	Age in years	Sex (woman or man)	Perceived knowledge of psilocybin	Concern for negative effects of psilocybin	Psilocybin should be legalized for recreational use	Psilocybin should be legalized for therapeutic use
Attitudes about psilocybin							
Age (in years)	-0.145						
Sex (woman or man)	-0.091	0.108					
Perceived knowledge of psilocybin	0.587^**^	-0.182^*^	-0.138				
Concern for negative effects of psilocybin	0.522^**^	0.097	0.138	0.410^**^			
Psilocybin should be legalized for recreational use	0.466^**^	0.002	-0.143	0.327^**^	0.372^**^		
Psilocybin should be legalized for therapeutic use	0.542^**^	-0.077	-0.039	0.236^**^	0.312^**^		

Regression model

Table 5 reports the results of the multiple regression analysis that was performed to identify variables that predict pharmacy students’ attitudes about medical psilocybin. Prior to performing the regression, multicollinearity testing was conducted. The independent variables were within acceptable variance inflation factors limits. Moreover, the three scales (self-assessed knowledge, concern, and attitudes about psilocybin) were normally distributed, implying the data did not deviate from normality enough to affect inference. The model produced a significant regression equation: (F(4, 122) = 40.575, p < 0.001), with an R2 = 0.571 (adjusted R2 = 0.557). Greater self-assessed knowledge about psilocybin, less concern over possible negative effects, greater belief in the decriminalization of psilocybin for recreational use, and greater belief in the decriminalization of psilocybin for therapeutic use were associated with more positive perceptions about medical psilocybin. The percentage of variance in the scores accounted for by the model was 57%.

**Table 5:** Multiple linear regression model for the dependent variable "attitudes about psilocybin" Predicted (dependent) variable: attitudes about psilocybin. * p < 0.05, ** p < 0.01.

Variable	B	SE	Beta (β)	t	p-value
Constant	13.711	4.498		3.048	0.003**
Perceived knowledge of medical psilocybin	0.793	0.139	0.372	5.705	0.000**
Concern for possible adverse effects of psilocybin	-0.465	0.170	-0.185	-2.731	0.007**
Belief in decriminalization (legalization) of psilocybin for recreational use	3.379	1.404	0.161	2.406	0.018**
Belief in decriminalization (legalization) of psilocybin for therapeutic use	10.821	1.977	0.354	5.473	0.000**

## Discussion

The current study aimed to understand how a small sample of pharmacy students perceived and were aware of the therapeutic potential of psilocybin. Specifically, greater self-assessed knowledge of psilocybin, more concern for possible negative effects from its use, and greater belief in the decriminalization of psilocybin for both recreational and therapeutic use were significant predictors of more positive attitudes about psilocybin in this sample of pharmacy students. Findings from this study support reports from previous studies with healthcare students regarding psilocybin-assisted therapy and medical cannabis [25,32-34]. This study also offers some insight into how pharmacy students think and feel about psilocybin as a treatment option, which could affect pharmacy education, and how psychedelic medications may be introduced into clinical settings in the future. However, in a study conducted with medical students regarding their attitudes toward psilocybin, more medical students surveyed reported that they thought psilocybin should be decriminalized for recreational use rather than for medical use [25]. Conversely, more pharmacy students reported in the current study that psilocybin should be decriminalized for therapeutic use, and less so for recreational use.

Self-assessed knowledge

The mean score for self-assessed knowledge of psilocybin in this sample was 4.30 on a scale of 1-6, with lower scores indicating lower levels of perceived knowledge. Although studies are limited, this finding is supported by previous studies on the level of knowledge about psilocybin among pharmacists [29] and medical students [25]. Given these findings, it could be conjectured that the lack of knowledge may be due to a deficiency in medical education regarding non-traditional treatments that have been summarized in the literature [38]. While there is little, if any, psilocybin training in pharmacy school, the majority (n = 151; 93.8%) of the participants in this study reported having a desire for psilocybin education while in school. Many participants reported they thought psilocybin would be beneficial to assist in treatments for anxiety (n = 105; 65%), depression (n = 96; 60%), and other psychiatric illnesses (n = 80; 50%). While some published reports that psilocybin might play an important role in the treatment of these disorders [6-10,12], pharmacy curricula that include a discussion of the uses, side effects, and legal and other limitations of psilocybin treatment may be useful.

Safety concerns

The study also examined the participants' concerns about the safety of psilocybin use and its potential negative consequences. The study's findings (i.e., less concern for possible adverse effects of psilocybin was associated with more positive attitudes about it overall) are consistent with previous studies suggesting that healthcare providers or trainees had concerns about psychedelic therapy [25-27]. However, acceptance was often moderated by concerns regarding safety and the lack of rigorous trials [26,27]. Nonetheless, some healthcare professionals have expressed confidence in psychedelic-assisted therapy within properly controlled settings [27].

Attitudes about psilocybin

The mean age of the participants was 24 years. As younger individuals, pharmacy students may be more likely to be aware of recent advances, be more accepting, and have more conversations about psychedelic therapy than their older counterparts. Furthermore, engaging pharmacy students in psilocybin education may allow for more receptivity to emerging treatment techniques in general, and a potential willingness to incorporate psychedelic medicines into patient care. The participants who believed in the decriminalization of psilocybin for either recreational or therapeutic use were more likely to have positive attitudes about psilocybin as a treatment, which is not surprising. Self-assessed knowledge also emerged as a predictor of attitudes toward psilocybin, as was seen in a previous study conducted with medical trainees. The students who reported higher levels of knowledge of medical psilocybin were less concerned about its potential negative effects [25]. While these findings are what one would expect, much more investigation of pharmacy students' (and pharmacists’) attitudes about psilocybin (including the possible benefits and risks of psilocybin itself) may be warranted.

Limitations

Several limitations of this study should be noted. Only one pharmacy program from a single institution with a relatively small sample size (less than 200 participants) may have contributed to self-selection bias; generalizations about all pharmacy students cannot be made. Also, the study was voluntary and may have an increased risk of sampling bias since students more likely to complete the survey may have a greater interest in psilocybin. Another limitation may be the focus on psilocybin and not on other psychedelic agents, which may carry differences in opinions. Moreover, the majority of the participants were in the pre-clinical phase of training; clinical students may have provided different responses. While these limitations caution generalizing these results to all pharmacy students, this is the first study to our knowledge that investigated psilocybin attitudes in this group and has the potential to add relevant pilot data to the existing body of literature.

## Conclusions

It appears that pharmacy students may lack information and training regarding psychedelic drugs such as psilocybin. However, many seem to have a desire to learn more about psilocybin. Pharmacy students’ overall attitudes may be driven by this desire to learn in addition to concern for adverse effects and legalization issues. Due to the dearth of published information regarding the knowledge and acceptance of psilocybin as a possible treatment option for patients suffering from particular illnesses, more research is needed regarding (1) the clinical benefits and risks of psilocybin therapy, (2) acceptance of psilocybin treatment in both pharmacy students and pharmacists, and (3) how pharmacy programs might leverage psilocybin content into the curriculum given legislative constraints. A focus for pharmacy educators and researchers might be to dovetail the core concepts of medication management into the curriculum and generate new evidence on the potential benefits and risks of psilocybin for certain health conditions.
